# A Multifunctional Reconfigurable Terahertz Metasurface Enabling Spin-Decoupled Logic Operations and Holography

**DOI:** 10.3390/ma18184362

**Published:** 2025-09-18

**Authors:** Zou Long, Zhengji Xu

**Affiliations:** 1 School of Microelectronics Science and Technology, Sun Yat-sen University, Zhuhai 519000, China; longz@mail2.sysu.edu.cn; 2 Guangdong Provincial Key Laboratory of Optoelectronic Information Processing Chips and Systems, Sun Yat-sen University, Zhuhai 519000, China

**Keywords:** reconfigurable metasurface, terahertz logic and holography, spin-decoupled CP control

## Abstract

We present a multifunctional, reconfigurable terahertz metasurface built from dual split-ring resonators combining photosensitive silicon and metallic elements. By hybridizing structural and Pancharatnam–Berry phase control, the device achieves spin-decoupled manipulation of circularly polarized wavefronts and an optical, light-intensity-driven reconfiguration mechanism. Using spatially encoded bifocal responses, we implement two two-input/two-output logic modules (OR-XOR and AND-NAND), and full-wave simulations verify the expected truth-table behaviors; additionally, a spin- and intensity-dependent hologram produces four distinct far-field images under different input conditions. At the selected working point (≈0.95 THz), the design exhibits a strong cross-polarization response (cross-polarized reflection amplitude > 0.7), demonstrating a viable route toward chip-scale, integrated terahertz logic and multifunctional imaging devices.

## 1. Introduction

With the rapid advancement of artificial intelligence, 6G communication systems, and optical computing technologies, there is an urgent demand for high-speed, energy-efficient, and integrable logic processing platforms beyond the limitations of conventional semiconductor electronics. Traditional electronic circuits are constrained by carrier mobility and thermal bottlenecks, making them less suitable for next-generation ultrafast computing. In contrast, all-optical logic systems offer significant advantages such as ultrahigh bandwidth, low latency, and inherent parallelism, making them highly promising candidates for future computing architectures [1,2,3,4].

In recent years, various strategies have been proposed to realize all-optical logic gates based on nonlinear effects, interferometric designs, and material tunability. While some successes have been achieved in demonstrating basic logic functions such as AND, OR, NOT, and XOR, these implementations often rely on bulky optical setups, high power consumption, or limited integration capability [5,6,7,8]. Moreover, many existing systems only support single-function logic operations and lack reconfigurability, thereby impeding the development of compact and scalable all-optical logic processors.

Metasurfaces, composed of subwavelength meta-atoms arranged on a planar surface, have revolutionized light manipulation by offering versatile control over the phase, amplitude, and polarization of electromagnetic waves [9,10,11,12]. Their ultrathin nature and design flexibility have enabled wide-ranging applications, including flat lenses [13,14], beam shaping [15,16], edge detection [17,18], color filtering [19,20], and holography [21,22,23]. More recently, metasurfaces have been employed to realize logic operations by encoding phase profiles or polarization-dependent responses into spatially varying structures [24]. Recent advances in metasurface theory and experiments—including analytical models for anisotropic meta-atoms and multipole-based analyses—have clarified how lattice-mediated interactions and higher-order modes shape scattering and resonance behavior [25,26,27]. In particular, lattice-induced multipole coupling and light–matter topology studies point to trapped high-Q modes and topology-based modal control that can be used to improve focusing contrast and holographic efficiency, complementing our transmission + Pancharatnam–Berry phase design with photo-induced switching [28,29]. Recent progress on tunable metadevices has shown practical reconfigurability in both optical and THz platforms [30,31]. A recent THz-metamaterial study demonstrating a cost-effective, temperature-tunable hybrid sensor on a single substrate further highlights the practical relevance of reconfigurable THz metasurface devices [32]. However, most reported metasurface-based logic gates remain limited to single-output, single-function operations and are rarely capable of integrating multiple functions such as logic and imaging within a unified platform.

A major challenge in metasurface-based logic devices lies in achieving multifunctionality, reconfigurability, and decoupled manipulation of polarization-dependent wavefronts. Existing logic metasurfaces generally exhibit spin-coupled responses, where the same structure induces opposite phase gradients for left- and right-circularly polarized (LCP/RCP) light [33,34,35,36,37], limiting their flexibility. Furthermore, few designs allow simultaneous control over logic operations and dynamic imaging, which is crucial for future chip-scale terahertz photonic processors. Addressing these gaps requires novel metasurface architectures that support independent phase encoding, optical switching, and dual-mode functionality.

In this work, we propose a multifunctional reconfigurable terahertz metasurface that enables spin-decoupled logic operations and holography by integrating photosensitive silicon and dual split-ring resonators (SRRs). By combining transmission phase and geometric phase modulation, our metasurface allows independent control of LCP and RCP waves under variable light intensity. We demonstrate two two-input, two-output parallel logic gate devices (OR-XOR and AND-NAND) based on polarization and illumination as inputs, with spatially encoded bifocal focusing used for logic state readout. In addition, we realize a dynamic metasurface hologram that displays four distinct far-field images depending on the input conditions. This work paves the way for compact, programmable terahertz systems that seamlessly integrate logic computing and information display functionalities.

## 2. Materials and Methods

To realize multifunctional logic operations and holography in the terahertz band, we designed a reconfigurable metasurface composed of dual concentric split-ring resonators (SRRs) integrating photosensitive silicon and metallic structures. The design utilizes light-intensity-sensitive switching mechanisms and polarization-selective responses to achieve independent spin-decoupled phase modulation. In this section, we first explain the working principles and structural composition of the metasurface. Then, we present the electromagnetic simulation setup, material parameters, and 2-bit phase encoding strategy used to implement spin-decoupled logic gates and holographic wavefront control.

**Figure 1:**
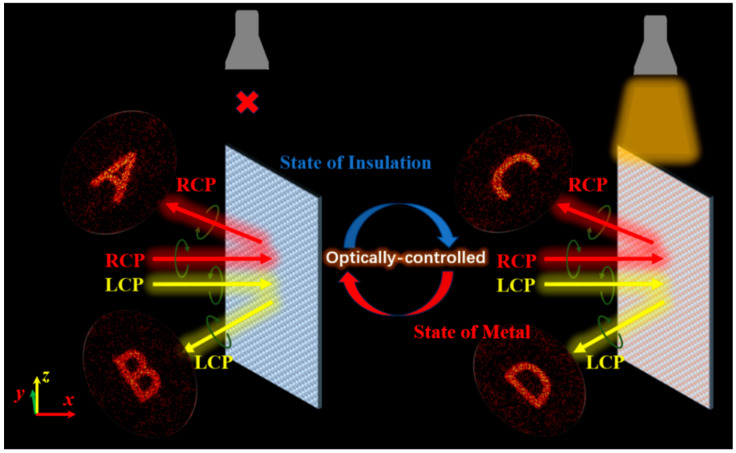
The schematic diagram of the functionalities of an optically reconfigurable metasurface.

To implement multifunctional terahertz wavefront control, we developed an optically reconfigurable metasurface capable of spin-decoupled responses. As illustrated in Figure 1, the working principle is based on the independent modulation of left-handed circularly polarized (LCP) and right-handed circularly polarized (RCP) waves under different illumination conditions. In the absence of light, the metasurface generates distinct holographic images “A” and “B” for LCP and RCP incidences, respectively. When illuminated, the response switches to holographic images “C” and “D” for the same polarizations. This dual-mode response mechanism enables four distinct output states through simple control of incident polarization and external light intensity.

**Figure 2:**
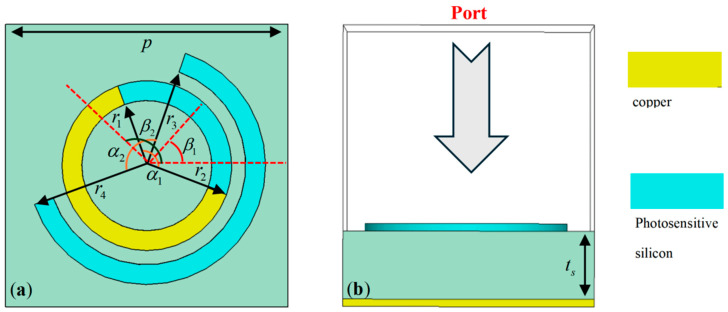
(**a**) Top view of the metasurface unit structure. (**b**) Side view of the metasurface unit structure.

Building on this principle, we designed two spin- and intensity-dependent logic gate devices. These devices take incident polarization and light intensity as dual inputs, and their outputs are encoded as focal intensities at predefined positions. The unit cell structure of the metasurface, shown in Figure 2, consists of a bottom metallic reflector, a middle polyimide dielectric spacer, and a top-layer resonator. The resonator comprises a larger split-ring resonator (SRR) made of photosensitive silicon and a smaller metallic SRR nested within it, with the void of the latter filled with complementary photosensitive silicon. This hybrid structure supports light-dependent switching between different resonant modes and enables polarization-specific phase modulation.

Photosensitive silicon is a light-responsive material widely used in the design of reconfigurable metasurfaces due to its tunable conductivity under optical excitation. When illuminated by an infrared light source centered at 800 nm, this material exhibits a marked increase in conductivity σ_Si_, which scales with the incident energy flux. In the absence of illumination, the silicon remains in a quasi-insulating state with σ_Si_ ≈ 20 S/m. Upon strong illumination, its conductivity increases dramatically to σ_Si_ ≈ 5 × 10^5^ S/m, rendering it highly conductive and effectively metallic [27].

In the proposed metasurface design, this light-dependent transition plays a crucial role in mode switching. Without illumination, the electromagnetic response is dominated by the smaller metallic split-ring resonator (SRR), while the photosensitive silicon remains inactive. Under strong illumination, the smaller metal SRR and the complementary silicon region form a closed conductive loop, suppressing resonant behavior due to symmetry or impedance mismatch. Simultaneously, the larger photosensitive silicon SRR becomes highly conductive and supports strong resonances, thereby taking over the response function. This enables a light-controlled switching mechanism between two resonant pathways, forming the basis of the reconfigurable spin-decoupled operation.

The unit cell structure of the metasurface has a periodicity of p = 100 μm. The inner and outer diameters of the larger SRR are r_i1_ = 35 μm and r_o1_ = 42 μm, respectively, while the outer and inner diameters of the smaller SRR are r_o2_ = 30 μm and r_i2_ = 23 μm, respectively. In the simulations, the metallic structures are made of copper with a conductivity of σ = 5.8 × 10^7^ S/m and a thickness of 3 μm. The dielectric layer is polyimide with a thickness of 27 μm, a dielectric constant of 3.5, and a loss tangent of 0.0027. The size and orientation of the opening in the larger SRR are denoted by α_1_ and θ_1_, respectively, while those for the smaller SRR are denoted by α_2_ and θ_2_. Notably, by adjusting the values of α_1_, θ_1_, α_2_, and θ_2_, it is possible to achieve independent control over LCP and RCP waves.

Traditional geometric phase metasurfaces achieve phase control of circularly polarized (CP) waves simply by rotating the resonator; however, there is phase coupling for CP waves with opposite spins. This means that the same metasurface array exhibits opposite phase gradients for LCP and RCP waves. In this work, we combine the principles of transmission phase and geometric phase. By altering the size of the openings and the rotation angles of the split rings, we achieve decoupled phase control for LCP and RCP waves.

Next, we analyze the theory behind spin-decoupling in metasurfaces. For any given meta-atom, its phase response can be divided into a transmission phase and a geometric phase. For the transmission phase, when the rotation angle of the unit structure is fixed, the phase response of linearly polarized light can be adjusted by altering the structural parameters of the meta-atom. In contrast, for the geometric phase, when a geometric phase meta-atom is rotated by an arbitrary angle θ, an additional phase shift of φ = ±2θ can be achieved. By combining these two phase responses, the meta-atom can independently control the phase response of orthogonal circularly polarized waves. The desired LCP and RCP phases can be expressed as follows [13]:


(1) $$\varphi_{LCP}=\phi_{LCP}^{Pro}+\phi_{LCP}^{PB}$$

(2) $$\varphi_{RCP}=\phi_{RCP}^{Pro}+\phi_{RCP}^{PB}$$

For the same meta-atom, the transmission phase generated by LCP and RCP is the same, and both can be expressed as follows:


(3) $$\phi_{LCP}^{Pro}=\phi_{RCP}^{Pro}=\phi^{Pro}$$

At the same time, the geometric phase corresponding to LCP and RCP can be described as follows:


(4) $$\phi_{LCP}^{PB}=-\phi_{RCP}^{PB}=2\theta$$

According to the above formula, once the $\varphi_{LCP}$ and $\varphi_{RCP}$ are determined, the corresponding transmission phase and geometric correspondence can also be determined:


(5) $$\phi^{Pro}=\frac{\phi_{LCP}+\phi_{RCP}}{2}$$

(6) $$\theta =\frac{\phi_{LCP}-\phi_{RCP}}{4}$$

Here, by varying the size and orientation of the openings in the split rings, we can obtain the corresponding transmission and geometric phases. To achieve CP wavefront control, we discretize the target phase range from 0° to 360° into four states and perform 2-bit encoding at intervals of 90. The digital codes “00”, “01”, “10”, and “11” are used to represent reflection phases of 0°, 90°, 180°, and 270°, respectively. Based on Equations (5) and (6), once the target phase distributions for the LCP and RCP reflected waves are known, the corresponding transmission phases and rotation angles can be calculated. Simulations can then be conducted to determine the appropriate structural parameters.

**Figure 3:**
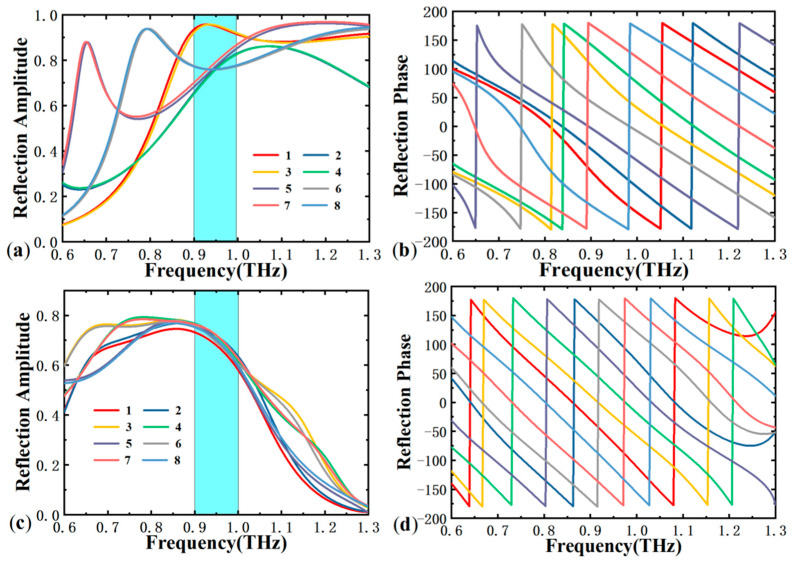
When the photosensitive silicon is in the insulating state: (**a**) the cross LP reflection amplitude of the meta-atom; (**b**) reflection phase. When the photosensitive silicon is in the metallic state: (**c**) the cross LP reflection amplitude of the meta-atom; (**d**) reflection phase.

To achieve 2-bit phase encoding for CP waves, the transmission phase of the meta-atom must reach a 3-bit encoding, meaning that the adjacent transmission phase gradients are spaced at 45° intervals. We present the cross-polarization reflection coefficients and reflection phases of photosensitive silicon in its insulating and metallic states, as shown in Figure 3. Figure 3a,b display the linearly polarized (LP) reflection amplitude and reflection phase of photosensitive silicon in the insulating state for the eight parameters required to achieve 2-bit encoding of CP waves. It can be observed that by altering the parameters of the smaller resonator, the reflection phase can cover the full 360° range across a wide bandwidth, maintaining uniform 45° intervals between the phase of the adjacent structures. Additionally, the reflection amplitude of these structures exceeds 0.7 in the 0.9–1.2 THz range, indicating high reflection efficiency. Figure 3c,d show the reflection amplitude and phase under the influence of the larger resonator when the photosensitive silicon is in a metallic state. It is evident that within the 0.75–0.95 THz frequency band, the reflection amplitude is greater than 0.7, and the phase maintains uniform 45° intervals across a wide bandwidth. Notably, in different states of the photosensitive silicon, the response amplitude of the metasurface unit remains above 0.7 at 0.95 THz, uniformly covering the full phase range. Therefore, we selected 0.95 THz as the operating frequency band for designing the 4-function metasurface. By adjusting light intensity and incident polarization, different wavefront controls can be achieved.

To achieve decoupled and independent control of orthogonal CP electromagnetic waves using a single metasurface, it is necessary to independently encode two CP incident waves within a single meta-atom. Therefore, by combining the 4 phase states of LCP waves and the 4 phase states of RCP waves, a total of 16 different structural parameters are required for the metasurface unit cells. Table 1 provides the resonator opening sizes and rotation angles corresponding to different CP phase encodings under no light conditions. Under illumination, the responsive resonator switches to the larger SRR. Table 2 presents the opening sizes and rotation angles of the larger SRR corresponding to different CP phase encodings under illuminated conditions.

**Figure 4:**
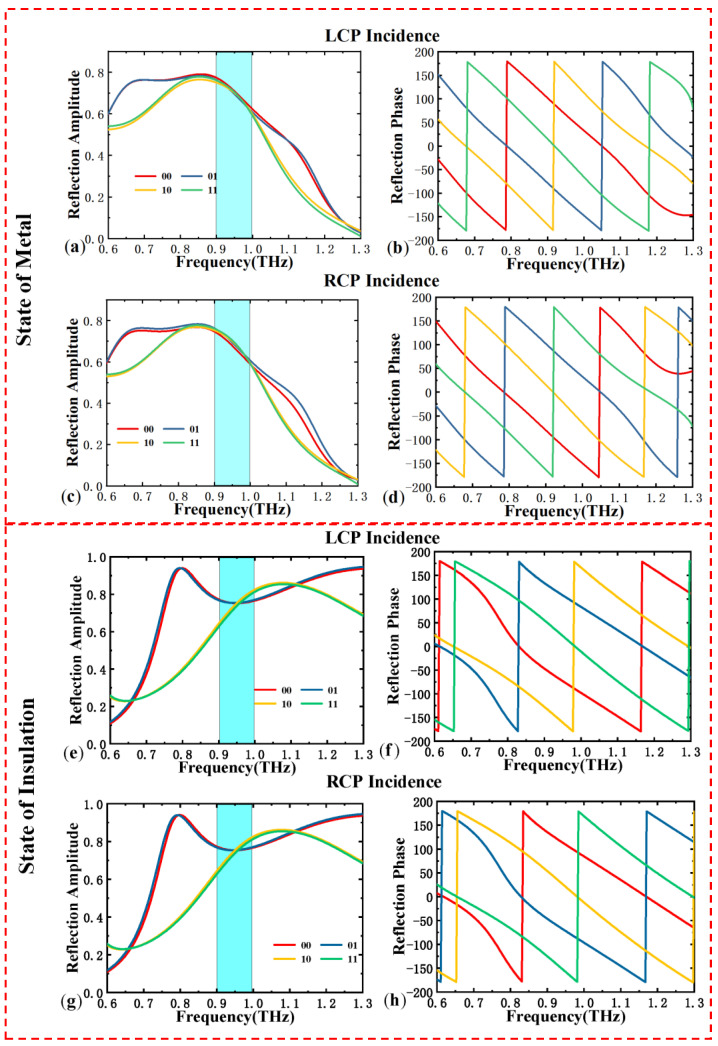
When the photosensitive silicon is in the metallic state: the four groups of unit structure (**a**) reflection amplitude of the incident LCP; (**b**) reflection phase; (**c**) reflection amplitude of the incident RCP; (**d**) reflection phase. When the photosensitive silicon is in the insulating state: the reflection amplitude of the four groups of unit structure (**e**) reflection amplitude of the incident LCP (**f**) reflection phase; (**g**) reflection amplitude of the incident RCP; (**h**) reflection phase.

To demonstrate that the proposed metasurface can achieve efficient spin-decoupling, we selected eight sets of parameters for which both LCP and RCP waves consistently remain in the states “00”, “01”, “10”, and “11”. We simulated the circular polarization reflection amplitude and phase under different light intensities. Figure 4a–d show the reflection coefficients for LCP and RCP incidence under no-light conditions. It can be observed that the reflection amplitudes and phases of LCP and RCP remain consistent, with phase intervals maintained at 90°, and the reflection amplitude exceeds 0.7 within the 0.9–1.2 THz range. Figure 4e–h display the reflection coefficients for the unit structure under LCP and RCP incidence in illuminated conditions. Similarly, under these conditions, the reflection amplitudes and phases of LCP and RCP remain consistent, with phase gradients matching the design expectations, and the reflection amplitude exceeds 0.7 within the 0.75–0.95 THz range. Therefore, these simulations demonstrate that with appropriately selected structural parameters, independent 2-bit spin encoding for circular polarization can be achieved.

## 3. Results and Discussion

All electromagnetic simulations were performed using CST Studio Suite (CST Microwave Studio).

### 3.1. Implementation of Logic Gates

**Figure 5:**
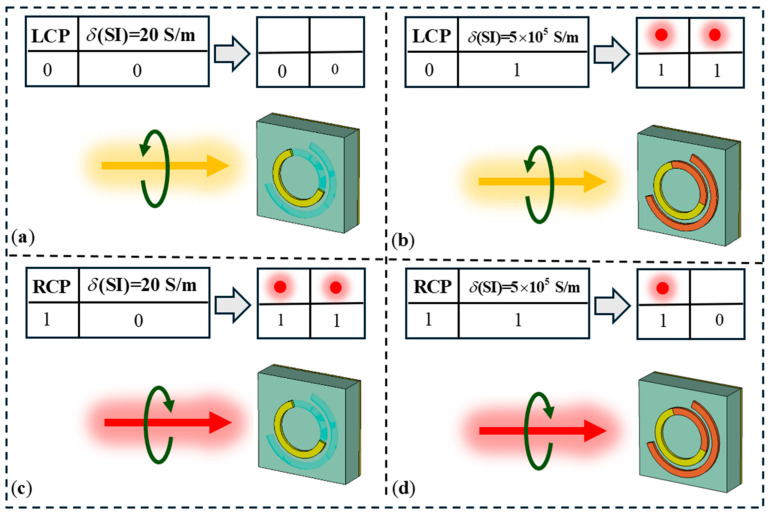
OR-XOR logic gate device schematic diagram. (**a**) Input (0,0), output (0,0). (**b**) Input (0,1), output (1,1). (**c**) Input (1,0), output (1,1). (**d**) Input (1,1), output (1,0).

Next, we designed two controllable bifocal metalenses to represent two-input, two-output parallel logic gate devices. The operational logic diagram is shown in Figure 5. The external light intensity and incident polarization serve as the two inputs for the metasurface array, where the illuminated condition is denoted as “1” and the no-light condition as “0.” LCP and RCP incidence are designated as the “0” and “1” input bits for the other input. For the OR-XOR logic device, the two inputs undergo OR and XOR logic operations, with the results displayed at focal points offset by x = −1000 μm and x = 1000 μm (represented as focal points A and B, respectively). Here, we use focal intensity to represent the output: if the focal intensity exceeds a certain threshold, it is denoted as “1”; if the intensity is below the threshold, it is denoted as “0”. For example, under no-light conditions with RCP incidence on the OR-XOR logic gate device, the input is (0, 1). After performing OR-XOR logic operations, the output is (1, 1), resulting in simultaneous focusing on both sides. Similarly, the AND-NAND logic device follows the same design principles. Figure 5b,c illustrate the input-output relationships for the two designed logic devices, forming the basis for our logic gate design.

Based on Figure 5b, the OR-XOR logic outputs include (0, 0), (1, 0), and (1, 1). We designed phase distributions corresponding to each of these three output states. For an off-axis lens, the phase distribution can be expressed as follows [33]:


(7) $$\Phi \left(x,y\right)=\frac{2\pi }{\lambda }\left(\sqrt{x^{2}+y^{2}+f^{2}}-f\right)+\Phi c\left(x,y\right)$$

Here, x and y are the coordinates of the metasurface unit structure in the xy plane, f is the preset focal length, and $\Phi c\left(x,y\right)$ represents the off-axis gradient phase. For the (1, 1) output, two symmetrically positioned lenses are generated. According to the addition principle, by overlaying two lenses with opposite off-axis gradients but the same focal length, we obtain the phase distribution for a bifocal lens. Conversely, for the (0, 0) output, no focal point exists in the reflection space; this is represented by a divergent phase, given by the following formula:


(8) $$\Phi \left(x,y\right)=-\frac{2\pi }{\lambda }(\sqrt{x^{2}+y^{2}+f^{2}}-f)$$

**Figure 6:**
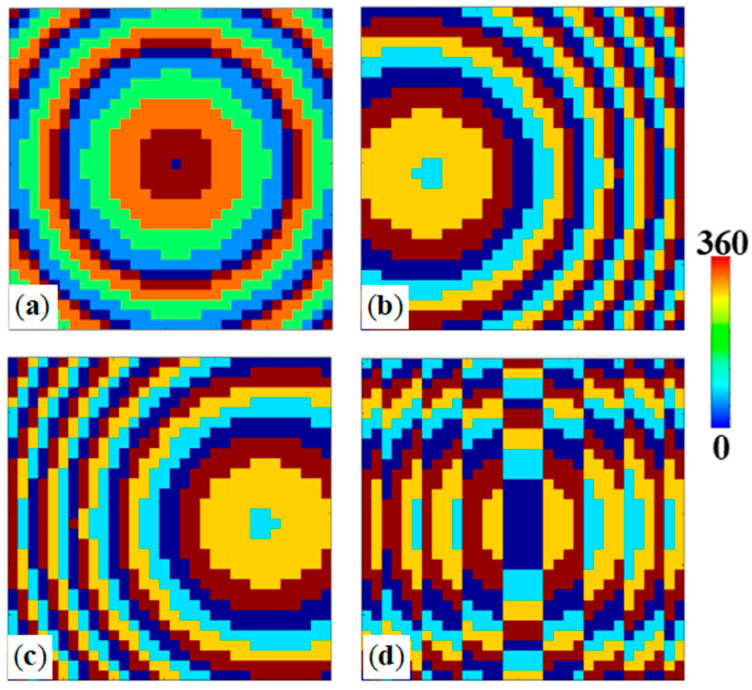
(**a**) Divergence phase compensation corresponding to output (0,0); (**b**) off-axis lens phase distribution corresponding to output (0,1); (**c**) off-axis lens phase distribution corresponding to output (1,0); (**d**) off-axis lens phase distribution corresponding to output (1,1).

**Figure 7:**
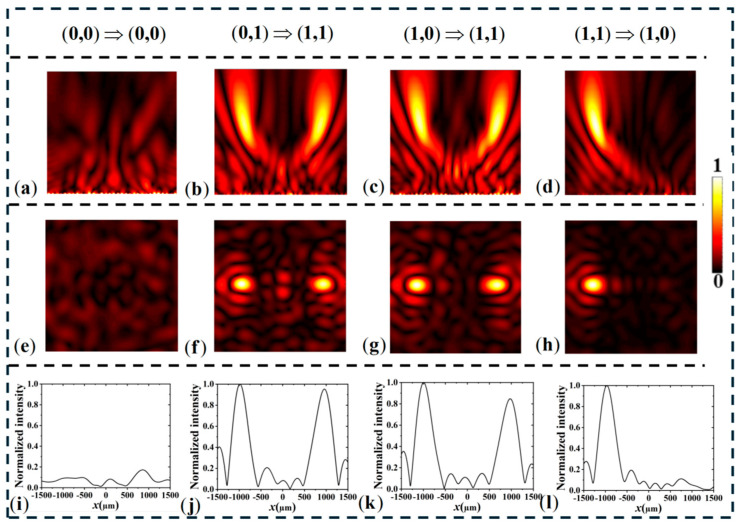
zx plane electric field distribution of simulated OR-XOR metasurface logic gate (**a**–**d**); (**e**–**h**) focal plane electric field distribution; (**i**–**l**) Normalized electric field distribution along the x-axis.

To verify the correctness of the design, we simulated the electric field distribution of the OR–XOR logic gate lens. As shown in Figure 6, the phase maps used to implement the spatially encoded bifocal readout for the logic gates. Panel (a) presents the divergence phase compensation corresponding to the output state (0,0), while panels (b)–(d) display off-axis lens phase distributions that steer the scattered field to the designated focal positions for outputs (0,1), (1,0), and (1,1), respectively. These calculated phase profiles were imposed on the metasurface unit-cell layout so that, under the appropriate input conditions, the scattered waves interfere constructively at the selected off-axis focal spots; the resulting focal intensity contrast is used to discriminate logical “1” and “0”. The effectiveness of these phase encodings is confirmed by the simulated far-field intensity patterns shown below. As shown in Figure 7, under no-light conditions, when a left-handed circularly polarized (LCP) plane wave is incident along the -z direction, it is converted into a divergent spherical wave by the metasurface, resulting in no focal point in the entire space. Therefore, the output can be considered as (0, 0). Under no-light conditions with RCP incidence, two focal points are generated on the reflective plane. In an illuminated environment with LCP excitation, two focal points also appear in the reflective space, corresponding to an output of (1, 1). For the fourth input scenario, under illuminated conditions with RCP incidence on the metasurface, a focal point is generated off-axis at x = −1000 μm, while no focal point appears on the symmetric side, corresponding to an output of (1, 0). Figure 7e–h show the normalized electric field distribution along the *x*-axis on the focal plane, consistent with the designed logic.

**Figure 8:**
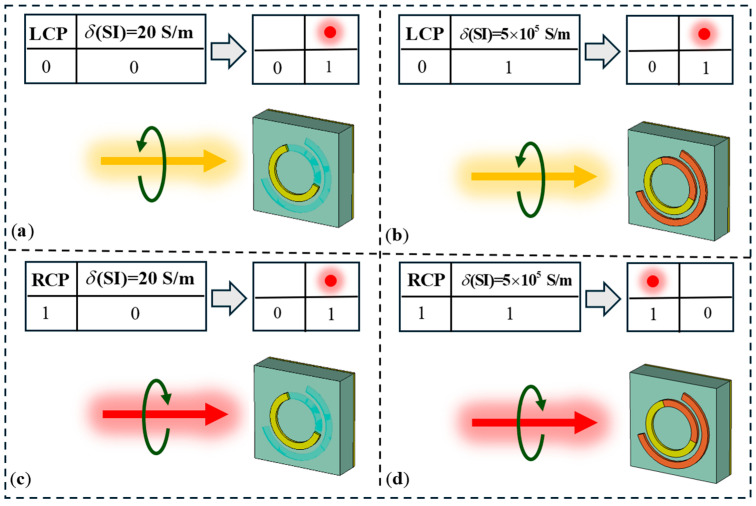
Schematic diagram of AND-NAND logic gate device. (**a**) Input (0,0), output (0,1). (**b**) Input (0,1), output (0,1). (**c**) Input (1,0), output (0,1). (**d**) Input (1,1), output (1,0).

**Figure 9:**
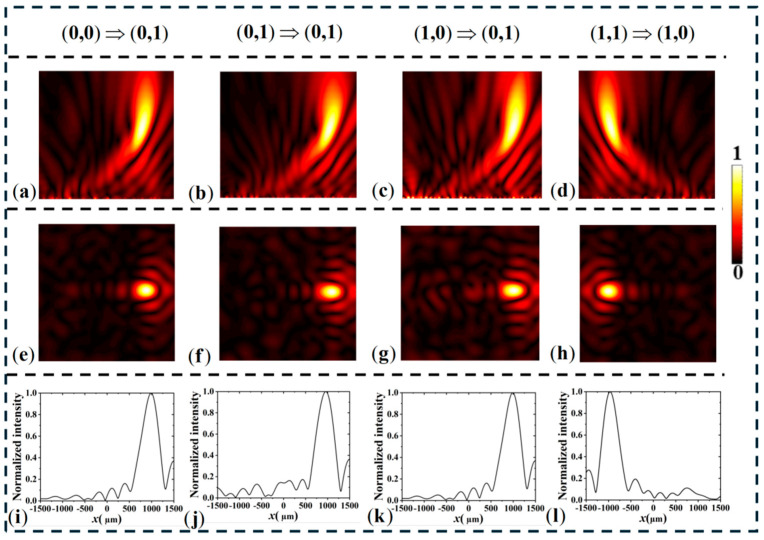
zx plane electric field distribution of simulated AND-NAND metasurface logic gate (**a**–**d**); (**e**–**h**) focal plane electric field distribution; (**i**–**l**) normalized electric field distribution along the x-axis.

To further demonstrate the logic operation capability of our designed reconfigurable metasurface, we developed another logic device based on the proposed unit structure: an AND-NAND logic gate. Similar to the OR-XOR design principle discussed earlier, the output results are represented by the focal intensities at two different positions. The required phase distributions for different inputs are shown in Figure 8. Similarly, we present the simulation results for the AND-NAND logic gate, as shown in Figure 9, which are consistent with the design.

### 3.2. Hologram

To demonstrate the multifunctional wavefront control capability of the designed unit structure, we have also developed a multifunctional metasurface hologram. Metasurface holography generates holographic images by calculating holographic interference patterns (including phase and amplitude information) and encoding them onto the surface structure. Compared to traditional holography, metasurface holography offers greater design freedom, allowing for optimized imaging algorithms that enhance image quality, eliminate unwanted diffraction components, improve imaging efficiency, and enable the creation of virtual objects that do not exist in reality. In this work, we utilize the proposed reconfigurable metasurface to achieve dynamic imaging in the reflection space by varying light illumination and incident polarization.

The diffraction between the metasurface and the imaging plane is a reversible process. Each unit of the metasurface array radiates an electromagnetic field outward, and according to the Rayleigh-Sommerfeld diffraction formula, the fields radiated by the units can be superimposed on the imaging plane. First, let us assume the radiated electric field of the metasurface is as follows [37]:


(9) $$U(x_{0},y_{0})=\frac{1}{j\lambda }\iint_{s}U(x,y)\cos\langle n,r\rangle \frac{\exp(jkr)}{r}dS$$

Here, $U(x_{0},y_{0})$ and U(x,y) represent the electric field distributions on the metasurface plane and the imaging plane, respectively; $\left(x_{0},y_{0}\right)$ and (x,y) are the position coordinates on the metasurface plane and the imaging plane. In the formula, r denotes the distance between any two points on the imaging plane and the metasurface, λ is the wavelength, and *zd* is the distance between the imaging plane and the metasurface. Since the reflection amplitude of the proposed metasurface is not controlled by the rotation angle of the resonator, it can only freely control the reflection phase. Here, the amplitude of $U(x_{0},y_{0})$ is set to 1, and the electric field on the imaging plane can be calculated as follows:


(10) $$U^{\prime }(x,y)=\frac{1}{j\lambda }\iint_{s_{0}}U(x_{0},y_{0})\cos\langle n,r\rangle \frac{\exp(-jkr)}{r}dS_{0}$$

We use the color intensity of a predefined pattern to replace $U^{\prime }(x,y)$ and use it as the input for the program. To obtain a high-resolution imaging pattern, the Gerchberg-Saxton (GS) iterative algorithm is employed to optimize the metasurface phase plate. Based on the optimized phase, the metasurface is modeled, and the electric field on the imaging plane is simulated and exported. The amplitude and phase information is extracted, and a hologram is constructed on this basis.

**Figure 10:**
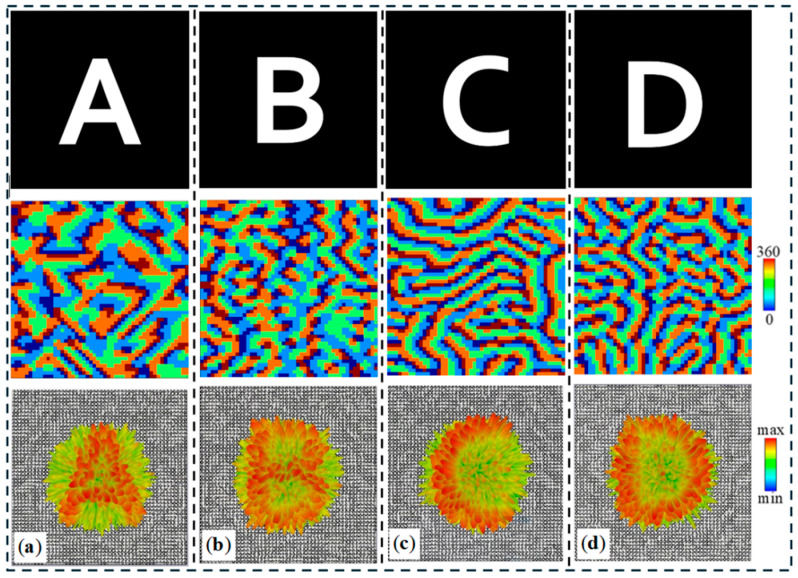
(**a**) Target image “A”. (**b**) Target image “B”. (**c**) Target image “C”. (**d**) The original image of the target image “D”, the surface phase distribution, and the imaging far-field distribution.

We used the shapes of the letters “A,” “B,” “C,” and “D” as the target patterns for metasurface imaging under four different input conditions. The designed metasurface consists of 50 × 50 unit cells, with an overall size of 5 mm × 5 mm. The phase distribution of the metasurface was optimized using the Gerchberg-Saxton (GS) algorithm, and the holographic phase distributions for the four patterns are shown in Figure 10a–d. In the simulations, different circularly polarized waves and varying external light intensities were used to excite the metasurface. Figure 10 shows the imaging results of the metasurface, where clear patterns of “A,” “B,” “C,” and “D” are displayed under different conditions, matching the target patterns. This demonstrates that the designed metasurface has excellent dynamic imaging capabilities.

## 4. Fabrication Process

**Figure 11:**
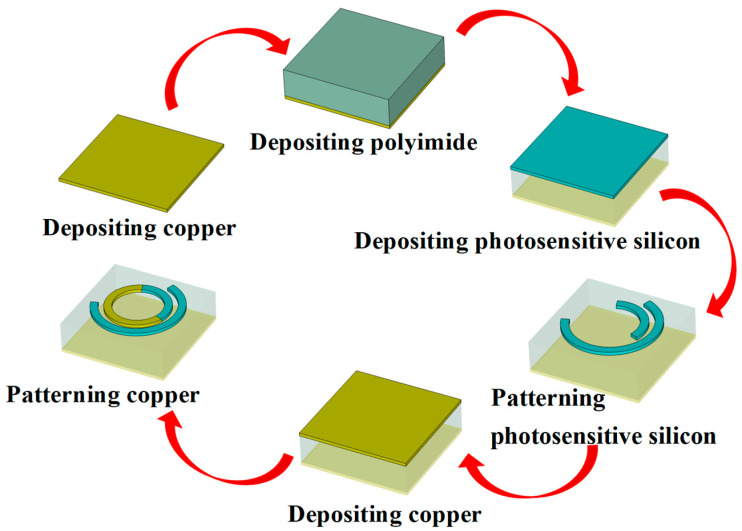
Fabrication process of metasurfaces based on photosensitive silicon for reconfigurable spin-decoupled terahertz metasurface.

To fabricate the proposed photosensitive silicon-based terahertz metasurface with dual split-ring resonators (SRRs), a multilayer microfabrication process can be employed using standard planar technologies. As shown in Figure 11, the process begins with the deposition of a reflective metal layer (e.g., 200 nm chromium followed by 3 μm gold) on a high-resistivity silicon or quartz substrate via magnetron sputtering. This metal layer serves as the bottom reflector to enhance the backward reflection in the terahertz band.

A 27 μm thick polyimide layer is then spin-coated onto the metal surface and thermally cured to act as a dielectric spacer. Next, the top-layer metasurface structure, consisting of a nested SRR pair, is fabricated. The larger outer SRR is composed of photosensitive silicon, which is deposited via plasma-enhanced chemical vapor deposition (PECVD) and patterned using standard photolithography followed by reactive ion etching (RIE).

Subsequently, the smaller inner metallic SRR is fabricated inside the cavity of the larger silicon SRR. This is achieved by spin-coating a photoresist layer, performing aligned photolithography, depositing a thin metal film (e.g., 100 nm gold), and conducting a lift-off process. Precise alignment between the two SRRs is crucial to ensure accurate polarization-dependent phase control.

Finally, optional passivation or anti-reflection coatings can be applied if required. The fabricated chip can then be diced and packaged for terahertz optical testing. This fabrication approach enables reliable realization of the proposed reconfigurable metasurface, ensuring structural fidelity and functional tunability under light illumination.

## 5. Conclusions

In summary, we have proposed a multifunctional reconfigurable terahertz metasurface that enables spin-decoupled wavefront control for advanced logic operations and dynamic holography. The metasurface integrates photosensitive silicon with dual split-ring resonators, allowing its response to be selectively tuned via light intensity and incident polarization. By combining transmission and geometric phase modulation, our design achieves independent phase control of circularly polarized waves, forming the basis for logic functionality. We demonstrated two two-input, two-output logic gate devices (OR–XOR and AND–NAND), as well as a polarization- and intensity-dependent holographic system capable of generating four distinct far-field images. This work highlights a viable approach for the development of compact, integrable terahertz systems with dual computing and imaging capabilities, paving the way for multifunctional metasurface-based platforms in future optical computing and communication technologies.

## Figures and Tables

**Table 1:** Resonator opening sizes and rotation angles corresponding to different CP phase encodings under no-light conditions.

	RCP	00	01	10	11
LCP					
00		α_1_ = 37° β_1_ = 45°	α_1_ = 85° β_1_ = 22.5°	α_1_ = 128° β_1_ = 0°	α_1_ = 170° β_1_ = 337.5°
01		α_1_ = 85° β_1_ = 67.5°	α_1_ = 128° β_1_ = 45°	α_1_ = 170° β_1_ = 22.5°	α_1_ = 37° β_1_ = 0°
10		α_1_ = 128° β_1_ = 90°	α_1_ = 170° β_1_ = 67.5°	α_1_ = 37° β_1_ = −45°	α_1_ = 85° β_1_ = −67.5°
11		α_1_ = 170° β_1_ = 112.5°	α_1_ = 37° β_1_ = 90°	α_1_ = 85° β_1_ = −22.5°	α_1_ = 128° β_1_ = −45°

**Table 2:** Resonator opening sizes and rotation angles corresponding to different CP phase encodings under illuminated conditions.

	RCP	00	01	10	11
LPC					
00		α_2_ = 20° β_2_ = 45°	α_2_ = 70° β_2_ = 22.5°	α_2_ = 130° β_2_ = 0°	α_2_ = 170° β_2_ = 337.5°
01		α_2_ = 70° β_2_ = 67.5°	α_2_ = 130° β_2_ = 45°	α_2_ = 170° β_2_ = 22.5°	α_2_ = 20° β_2_ = 0°
10		α_2_ = 130° β_2_ = 90°	α = 170° β_2_ = 67.5°	α_2_ = 20° β_2_ = −45°	α_2_ = 70° β_2_ = −67.5°
11		α_2_ = 170° β_2_ = 112.5°	α_2_ = 20° β_2_ = 90°	α_2_ = 70° β_2_ = −22.5°	α_2_ = 130° β_2_ = −45°

## Data Availability

The original contributions presented in this study are included in the article. Further inquiries can be directed to the corresponding author.
